# A Direct and Non-Singular UKF Approach Using Euler Angle Kinematics for Integrated Navigation Systems

**DOI:** 10.3390/s16091415

**Published:** 2016-09-02

**Authors:** Changyan Ran, Xianghong Cheng

**Affiliations:** 1 School of Instrument Science & Engineering, Southeast University, Nanjing 210096, China; ranchangyan@126.com; 2 College of Computer and Information Technology, Three Gorges University, Yichang 443000, China; 3 Key Laboratory of Micro-Inertial Instrument and Advanced Navigation Technology, Ministry of Education, Southeast University, Nanjing 210096, China

**Keywords:** strapdown inertial navigation system (SINS), unscented Kalman filters (UKF), integrated navigation, Euler angle kinematics, singularity

## Abstract

This paper presents a direct and non-singular approach based on an unscented Kalman filter (UKF) for the integration of strapdown inertial navigation systems (SINSs) with the aid of velocity. The state vector includes velocity and Euler angles, and the system model contains Euler angle kinematics equations. The measured velocity in the body frame is used as the filter measurement. The quaternion nonlinear equality constraint is eliminated, and the cross-noise problem is overcome. The filter model is simple and easy to apply without linearization. Data fusion is performed by an UKF, which directly estimates and outputs the navigation information. There is no need to process navigation computation and error correction separately because the navigation computation is completed synchronously during the filter time updating. In addition, the singularities are avoided with the help of the dual-Euler method. The performance of the proposed approach is verified by road test data from a land vehicle equipped with an odometer aided SINS, and a singularity turntable test is conducted using three-axis turntable test data. The results show that the proposed approach can achieve higher navigation accuracy than the commonly-used indirect approach, and the singularities can be efficiently removed as the result of dual-Euler method.

## 1. Introduction

Integrated navigation systems, in which two or more navigation systems are combined to yield greater precision than any single component system operating in isolation, are usually employed in practice. It is common to use strapdown inertial navigation systems (SINSs) to provide data with good short term accuracy, while other sensors are employed to provide good long term stability.

The variety of modern navigation aids now available is extensive, but external navigation aids are limited for military and underwater applications where accurate navigation is required without the help of external information, such as GPS, which may be disturbed or blocked. In such circumstances, the aid of velocity is popular because it is more reliable and accessible. As a result, the integration of SINSs with velocity aid has become a standard approach and received much attention. Velocity observations may be provided by velocimeters [1,2], e.g., odometers are used for land vehicles [3,4], and Doppler velocity logs (DVLs) are generally mounted on the shell for autonomous underwater vehicles (AUVs) [5,6]. Given the popularity of the integration of SINS/odometer and SINS/DVL, this paper focuses on the SINSs aided by the external information about the vehicle velocity in the body frame due to the full self-contained characteristic, although these are limited cases in the integrated navigation system where only velocity aids are used.

Depending on whether the navigation error variables are utilized as the states, the approach for information combination of integrated navigation systems is broadly categorized as a direct or indirect type [4]. The well-known approaches are mostly indirect for the integration of SINS/odometer and SINS/DVL, where SINS error models are employed. The filter states are navigation errors defined as the differences between the calculated values and the true values. The filter measurements are the differences between external aid measurements projected into the navigation frame and the corresponding quantities derived from SINSs. Suitable filters are used to estimate the errors in order to correct the navigation information calculated by the SINSs.

The typically indirect approaches from extensive publications [3,4,5,6,7] employ linear Kalman filters (KFs), where the SINS error models are linearized with the assumption of small navigation errors. These KF-based techniques suffer from divergence due to the linearization approximations and system mismodeling. Liu et al. [6] studied the cross-noise problem and introduced a cross-noise term into the predicted error covariance, the cross-covariance and the innovation covariance in their proposed Kalman filter whose algorithm is a little complex.

Another kind of indirect approaches employ nonlinear filters, where only the measurement models are nonlinear [8], only the SINS error models are nonlinear [9,10,11], or both are nonlinear [12]. The nonlinearity limits the application of the linear Kalman filters. The unscented Kalman filter (UKF) [13] is used to do the integration of GPS/INS/Odometer/Inclinometer [8]. A nonlinear SINS error model with large heading errors is used [10,11]. A modified UKF using simplex sigma point sets [14] and an adaptive UKF are employed for the integration of low-cost SINS/GPS [10,11]. An adaptive UKF and a nonlinear SINS error model with three large Euler angle errors are used for the integration of SINS/DVL [9,12].

In contrast, direct approaches for data fusion of integrated navigation systems use navigation information instead of the errors as the filter states. Some are based on linear Kalman filters [15,16,17], while others are based on nonlinear filters [18,19,20,21,22,23,24,25,26]. Since the quaternion kinematics equation is linear, most of direct approaches employ the attitude quaternion as the state [16,17,18,19,20,21,22,23]. Quaternion representation has four components with one redundant parameter, and a normalization constraint has to be addressed in filtering algorithms. However, the KFs and UKFs are not designed to preserve the unit-norm property of the quaternion, and consequently the filter algorithm should be revised. The problem of Kalman filtering with nonlinear equality constraints is discussed in detail [23].

A direct Kalman filtering approach is presented in [15], where a direct GPS/INS model is initially introduced with the states including position and velocity, but not the attitude. A novel quaternion KF with four attitude quaternion components as the states is presented for spacecraft attitude estimation [16], and it is further applied to the initial orientation of SINS/odometer [17]. A linear quaternion pseudo-measurement equation is derived, and a linear system model and a linear measurement model are constructed to eliminate the linearization as in nonlinear error models. The expressions for the covariance matrices of the state-dependent system noises are very complex.

A quaternion UKF is applied to the integration of low-cost IMU/GPS/digital compass [18], and the quaternion constraint is not considered in the filtering algorithm. The quaternion constraint is handled in the transportation of the sigma points and the updates of the mean by quaternion multiplication in quaternion UKFs [19,20,21]. Another quaternion sigma-point KF maintains numerical stability and the unity norm of the quaternion by adding a small Lagrange multiplier term [22]. A multiplicative quaternion-error approach, in which an unconstrained three-component attitude-error vector is employed among the states, is presented to guarantee that quaternion normalization is maintained in the filter [24]. The states of the UKF are partly navigation errors, thus it can be thought of as a semi-direct approach.

Euler angles are used among the states [1,2,25,26]. Bristeau et al. focus on low cost setups, incorporating a MEMS IMU, a velocimeter and an altimeter. The filter models are linear, and a collection of filters are designed during different trajectories according observability analysis [1,2]. Georgy et al. propose a mixture particle filter to perform the integration of reduced SINS/odometer/map data/GPS. The inertial sensors consist of one vertical MEMS-based single-axis gyro and two horizontal accelerometers, thus the system is not a typical aided SINS. Moreover, the method targets better modeling of the low-cost gyro and the processing of the outages of GPS [25]. An asynchronous direct Kalman filter (ADKF) approach is presented for underwater integrated navigation system to improve the performance of the popular indirect Kalman filter structure [26]. The system and the measurement equations are nonlinear, and an extended Kalman filter (EKF) is used to do the data fusion. During the filtering process, the system and measurement equations are linearized analytically by evaluating the Jacobian matrices, which is very complex and difficult to derive the analytic expressions. As we all know, an EKF uses the first-order Taylor series to approximate the nonlinear processes. The predict-update cycle remains identical to the KF. The EKF tends to underestimate the variance of the states, which can lead to large inaccuracies in strong nonlinearity.

This paper aims to develop a novel general direct data fusion approach using an UKF for the SINS, which navigates the vehicle in the local geographic frame with the aid of a velocity sensor mounted on the body. This approach is developed to overcome the aforementioned limitations in indirect approaches and in quaternion UKFs. The proposed approach is based on Euler angle kinematics equations and uses the Euler angles among the states. Since Euler angles are independent of each other, the constraint in the above mentioned quaternion UKFs no longer exists. The predicted mean and covariance can be directly computed using the standard UKF equations. The velocity in the body frame is used as the filter measurements without the velocity projection from the body frame to the local-level frame as in indirect approaches, so the cross-noise problem is avoided. The system equations employ navigation equations, Euler angle kinematics equations and the inertial sensor measurement bias differential equations. The UKF is used to estimate the states and outputs the navigation information directly. The UKF choses a number of points so that their mean and covariance can approximate statistical linearization to replace the analytical linearization of the EKF, as a result, the complex Jacobian matrices are not necessary. The proposed approach combines the navigation computation with the state estimation, and there is no need to process navigation computation and error revision separately. The performance is verified by road test data obtained from a land vehicle equipped with an odometer aided SINS. Meanwhile, the singularity problem which exists in the Euler angles in the direct approach is solved by means of the dual-Euler method. The positive and passive Euler angle direct approaches run alternately in terms of the switch flag to remove the singularities, so the proposed approach is non-singular. A singularity turntable test is conducted using three-axis turntable test data.

The rest of this paper is organized as follows: the system equations and the measurement equations are established in Section 2; the UKF algorithm is presented in detail in Section 3; the experimental results are presented in Section 4 to endorse the performance of this novel data fusion approach; a turntable test is shown in Section 5 to examine the capability of dual-Euler method to avoid the singularity; concluding remarks are provided in Section 6.

## 2. System Model and Measurement Model

The local geographic navigation frame mechanization is used. The main coordinate frames used in this paper different from other references are defined as follows: the body frame (*b*-frame) coordinate system of the vehicle is the orthogonal reference frame aligned with the inertial measurement unit (IMU) axes, which is defined with *x* axis along the transversal direction right, *y* axis along the longitudinal direction forward, and *z* axis along the vertical direction upward, completing a right-handed system (right-forward-upward, RFU). The local geographic frame is used as the navigation frame (*n*-frame), which is the orthogonal reference frame aligned with east-north-up (ENU) geodetic axes.

Velocity measurements in the *b*-frame are used to aid the on-board navigation system. Suitable measurements may be provided by odometers or DVLs. In order to provide estimates of the attitude, the velocity and the position in the on-board inertial navigation system, a ten state UKF is designed.

**Figure 1:**
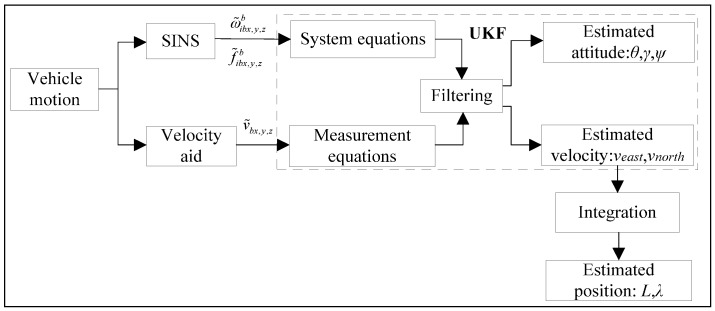
Diagram of the direct data fusion approach for a *b*-frame velocity aided SINS.

A block diagram representation of the direct data fusion approach for a *b*-frame velocity aided SINS is shown in Figure 1.

Where ${\tilde{f}}_{ibx,y,z}^{b}$ and ${\tilde{\omega }}_{ibx,y,z}^{b}$ are the accelerometer and gyro measurements along *x*, *y*, *z*-axis of body, *θ*, *γ*, *ψ* are the pitch, roll and heading, respectively, *v_east_* and *v_north_* are the east and north vehicle velocities with respect to the earth, respectively, *L* is the geographic latitude, *λ* is the longitude, ${\tilde{v}}_{bx,y,z}$ are the velocity aid measurements along *x*, *y*, *z*-axis of body. In Figure 1, the vehicle motion changes the outputs of the inertial sensors and the aid, which are inputs of the system equations and measurement equations of the UKF, respectively. The UKF compares the *b*-frame velocity derived from aid outputs with the corresponding quantity estimated from the SINS, and estimates the inertial sensor biases, the attitude and the velocity in the *n*-frame. The *n*-frame velocity is then integrated into the position. The associated nonlinear system equations and measurement equations are described in the sections that follow.

### 2.1. System Model

The system process comprises the SINS mechanization and the inertial sensor error modeling. For a terrestrial navigation system operating in the local geographic reference frame, the navigation equation may be expressed as:

(1) $${\dot{v}}_{en}^{n}=f^{n}-\left(2\omega_{ie}^{n}+\omega_{en}^{n}\right)\times v_{en}^{n}+g^{n}$$

where $v_{en}^{n}$ represents the velocity with respect to the earth expressed in the *n*-frame defined by the directions of east, north , and the local vertical in component form:

(2) $$v_{en}^{n}={\left[v_{east},v_{north},v_{up}\right]}^{T}$$

where *v_up_* is the vertical component of vehicle velocity with respect to the Earth, [•]*^T^* is the transpose of [•].

The specific force vector expressed in the *n*-frame ***f****^n^* is given by:

(3) $$f^{n}=C_{b}^{n}f_{ib}^{b}$$

(4) $$C_{b}^{n}={\left(C_{n}^{b}\right)}^{T}$$

where $C_{b}^{n}$ is the direction cosine matrix (DCM) representing the rotational transform of vectors from the *b*-frame to the *n*-frame. Rotating by the sequence of heading, pitch and roll leads to $C_{b}^{n}$, and the three successive rotations may be expressed mathematically as three separate direction cosine matrices defined as ***C****_ψ_*, ***C****_θ_*, and ***C****_γ_*, respectively. *ψ* ∈ [−π,+π], and it is defined with a positive direction anticlockwise from the north. Therefore, $C_{n}^{b}$ is expressed as the product of these three separate transformations as follows:

(5) $$\begin{array}{l} C_{n}^{b}=C_{\gamma }C_{\theta }C_{\psi }=\left[\begin{array}{ccc} \cos\gamma & 0 & -\sin\gamma \\ 0 & 1 & 0\\ \sin\gamma & 0 & \cos\gamma \end{array}\right]\left[\begin{array}{ccc} 1 & 0 & 0\\ 0 & \cos\theta & \sin\theta \\ 0 & -\sin\theta & \cos\theta \end{array}\right]\left[\begin{array}{ccc} \cos\psi & \sin\psi & 0\\ -\sin\psi & \cos\psi & 0\\ 0 & 0 & 1 \end{array}\right]\\ =\left[\begin{array}{ccc} \cos\gamma \cos\psi -\sin\theta \sin\gamma \sin\psi & \cos\gamma \sin\psi +\sin\theta \sin\gamma \cos\psi & -\cos\theta \sin\gamma \\ -\cos\theta \sin\psi & \cos\theta \cos\psi & \sin\theta \\ \sin\gamma \cos\psi +\sin\theta \cos\gamma \sin\psi & \sin\gamma \sin\psi -\sin\theta \cos\gamma \cos\psi & \cos\theta \cos\gamma \end{array}\right] \end{array}$$

The true specific force vector $f_{ib}^{b}$ is given by:

(6) $$f_{ib}^{b}={\left[{\tilde{f}}_{ibx}^{b}-\delta f_{ibx}^{b},{\tilde{f}}_{iby}^{b}-\delta f_{iby}^{b},{\tilde{f}}_{ibz}^{b}-\delta f_{ibz}^{b}\right]}^{T}$$

where $\delta f_{ibx,y,z}^{b}$ are the accelerometer measurement errors along *x*, *y*, *z*-axis of body.

The other terms in Equation (1) are given by:

(7) $$\omega_{ie}^{n}={\left[0,\omega_{ie}\cos L,\omega_{ie}\sin L\right]}^{T}$$

(8) $$\omega_{en}^{n}={\left[-\frac{v_{north}}{r_{M}},\frac{v_{east}}{r_{N}},\frac{v_{east}}{r_{N}}\tan L\right]}^{T}$$

(9) $$r_{M}=r_{q}(1-2\rho +3\rho \sin^{2}L)$$

(10) $$r_{N}=r_{q}(\rho \sin^{2}L+1)$$

(11) $$g^{n}={\left[\begin{array}{ccc} 0, & 0, & -g \end{array}\right]}^{T}$$

where $\omega_{ie}^{n}$ is the Earth rotation rate with respect to the inertial frame expressed in the *n*-frame, and $\omega_{en}^{n}$ represents the turn rate of the *n*-frame with respect to the Earth-fixed frame expressed in the *n*-frame, i.e., the transport rate, $\omega_{ie}$ is the Earth rotation rate, *r_M_* and *r_N_* are the meridian and transverse radius of curvature of the Earth’s reference ellipsoid, respectively, *r_q_* is the length of the semi-major axis, *ρ* is the flattening of the ellipsoid, and *g* is the local Earth’s gravitational acceleration.

A large number of parameterizations exist in the literature for attitude matrix. Reference [27] contains a complete survey of attitude representations. DCM, Euler angles, the quaternion, Rodrigues parameters (RP), and modified Rodrigues parameters (MRP) are the most popular ones. A generalization of the Rodrigues parameters (GRP) is presented in [28]. The GRP can be used to construct a set of three symmetric stereographic parameters, or to construct a set of three asymmetric stereographic parameters. The RP and MRP can be considered a special case of the general symmetric stereographic parameters. The RP, MRP and GRP are all derived from the quaternion by means of a stereographic projection of the four-dimensional unit sphere onto a three-dimensional hyperplane. A redundant parameter of the quaternion is removed, and the amount of calculation is reduced. Every parameterization has its own advantages and disadvantages. The main properties of some parameterizations of attitude rotational matrix are listed in [29].

The DCM is of little use except for analytical studies and transformation of vectors largely because of its nine dimensions with six redundant parameters. The quaternion representation is the subject of many literatures and frequently uses in practices, but the quaternion constraint makes undesirable implementation of the quaternion UKF as mentioned before. The Euler angles, RP, MRP and GRP have three uncorrelated parameters sufficient to describe a general rotation. They all have the minimum dimensions for attitude representation, and the Euler angle representation is the most famous and the most commonly used. The RP, MRP and GRP are often used in attitude determination and control for spinning bodies like satellites, where the vehicles rotate mainly about a certain axis, i.e., the vehicles have regular angular motion parameters. The Euler angles are easiest to visualize geometric recipe to describe the motion of any vehicle with respect to a reference frame by three successive rotation angles. There are twelve Euler angle sequences associated with twelve representations of the DCM.

As we all know, any set of three parameters has singularities. Different three-parameter sets distinguish themselves by having their singularities at different orientations. The RP, MRP, GRP have their singular point determined by different stereographic projections. The singularity for the classical RP and MRP is ±π and ±2π respectively with respect to rotation angles. The singularity for the symmetric GRP can be anywhere between a principal rotation of 0 and 2π, and the singularity for the asymmetric GRP is determined by both a principal angle and an axis of rotation. Different rotation sequences have different singularities for Euler angle sets. The 3-2-1 Euler angle set has a singularity at *θ =* ±π/2. The problem of how to avoid singularity associated with a three-element attitude parameterization has been well studied in the literatures. The approaches are mainly based on the method of sequential rotations (MSR), which removes the singularity by switching from singular sets to non-singular sets at the singularity for RP, MRP and Euler angles. Two applications are presented in detail to show how to avoid the singularity when using RP and MRP in [30]. And this problem is discussed for GRP in [28] and for Euler angles in [29,31,32].

The Euler angle representation is perhaps one of the simplest techniques in terms of physical appreciation. Most of all, the three Euler angles are uncorrelated. Therefore, when the Euler angles are used as the filter states, there is no extra change in the filtering.

Although the Euler angle representation suffers from the so-called singularity, the tests covered in this paper, conducted in a land vehicle with little horizontal attitude change, will not suffer from it. The Euler angle representation is suitable for this case because the calculated attitude angles have high accuracy when *θ* is close to 0 or ±π. The Euler angle sequence 3-2-1 is adopted, as indicated by Equation (5). If operation *θ→*±π/2 is desired singularity could occur, and in this circumstance an alternative set of Euler angles, e.g., the 3-1-2 Euler angle set in which the rotation sequence is heading-roll-pitch, can be employed to remove the singularity [27,29,31,32]. The best conversion angles are ±π/4 and ±3π/4, which are discussed and examined in [32,33]. Moreover, the Euler angles are very intuitive to work with, making the final filtering simpler. The Euler angle representation, like the above mentioned three-parameter representation, is employed in this paper.

The propagation of the Euler angles with time is governed by the following differential equations:

(12) $$\left[\begin{array}{c} \dot{\theta }\\ \dot{\gamma }\\ \dot{\psi } \end{array}\right]=\frac{1}{\cos\theta }\left[\begin{array}{ccc} \cos\theta \cos\gamma & 0 & \sin\gamma \cos\theta \\ \sin\theta \sin\gamma & \cos\theta & -\cos\gamma \sin\theta \\ -\sin\gamma & 0 & \cos\gamma \end{array}\right]\omega_{nb}^{b}$$

where $\omega_{nb}^{b}$ is angular velocity of the *b*-frame relative to the *n*-frame expressed in the b-frame. The solutions of the $\dot{\gamma }$ and $\dot{\psi }$ equations become indeterminate at the singularities where θ = ±π/2. $\omega_{nb}^{b}$ is given by:

(13) $$\omega_{nb}^{b}=\omega_{ib}^{b}-C_{n}^{b}(\omega_{ie}^{n}+\omega_{en}^{n})$$

The true angular rate vector $\omega_{ib}^{b}$ is given by:

(14) $$\omega_{ib}^{b}={\left[{\tilde{\omega }}_{ibx}^{b}-\delta \omega_{ibx}^{b},{\tilde{\omega }}_{iby}^{b}-\delta \omega_{iby}^{b},{\tilde{\omega }}_{ibz}^{b}-\delta \omega_{ibz}^{b}\right]}^{T}$$

where $\delta \omega_{ibx,y,z}^{b}$ are the gyro output errors along *x*, *y*, *z*-axis of body. Equation (12) is known as the Euler angle kinematics equation. The way to obtain the Euler angle rates from the angular velocity is similar to the derivation of Equation (294) in [27], but Equation (12) is not identical to it because of the definition of the *b*-frame, *n*-frame and the Euler angles.

The gyro measurement errors $\delta \omega_{ibx,y,z}^{b}$ in Equation (14) and the accelerometer measurement errors $\delta f_{ibx,y,z}^{b}$ in Equation (6) are modeled as the sum of a Gaussian white noise and a bias expressed in the *b*-frame. Therefore, the IMU measurement errors are given by:

(15) $$\delta \omega_{ibx}^{b}=\varepsilon_{gx}+w_{gx},\delta \omega_{iby}^{b}=\varepsilon_{gy}+w_{gy},\delta \omega_{ibz}^{b}=\varepsilon_{gz}+w_{gz}$$

(16) $$\delta f_{ibx}^{b}=\nabla_{ax}+w_{ax},\delta f_{iby}^{b}=\nabla_{ay}+w_{ay},\delta f_{ibz}^{b}=\nabla_{az}+w_{az}$$

where *ε_gx,y,z_* and *w_gx,y,z_* are the gyro bias and noise components along *x*, *y*, *z*-axis of body respectively, ∇*_ax,y,z_* and *w_ax,y,z_* are the accelerometer bias and noise components along *x*, *y*, *z*-axis of body respectively. With the assumption that the gyro and accelerometer biases can be represented as random constants, inertial sensor bias dynamics may be given by the trivial differential equations as follows:

(17) $${\dot{\nabla }}_{ax}=0,{\dot{\nabla }}_{ay}=0,{\dot{\nabla }}_{az}=0,{\dot{\varepsilon }}_{gx}=0,{\dot{\varepsilon }}_{gy}=0,{\dot{\varepsilon }}_{gz}=0$$

East and north velocities, all three Euler angles, and the inertial sensor biases are chosen as the states. The state vector can be expressed in component form as:

(18) $$x={\left[v_{east},v_{north},\theta ,\gamma ,\psi ,\nabla_{ax},\nabla_{ay},\varepsilon_{gx},\varepsilon_{gy},\varepsilon_{gz}\right]}^{T}$$

where ***x*** is the ten-dimensional state vector.

The system noise vector that represents the inertial sensor noise can be written as:

(19) $$w={\left[w_{ax},w_{ay},w_{gx},w_{gy},w_{gz},0,0,0,0,0\right]}^{T}$$

where ***w*** is the ten-dimensional system noise vector, which is modeled as Gaussian white noise. ***w*** has zero mean and is normally distributed (Gaussian) with a power spectral density of ***Q***. ***Q*** is a 10 × 10 diagonal matrix, the elements of which are selected in accordance with the anticipated level of inertial sensor measurement noise.

According to the selected state vector in Equation (18) and the system noise vector in Equation (19), Equations (1), (12) and (17) can be combined and expressed in state space form as:

(20) $$\dot{x}=F_{c}(x)+G(x)w$$

where ***F_c_***(***x***) is the nonlinear state transfer function, and ***G***(***x***) is the system noise input function. ***F_c_***(***x***) is a 10 × 1 function matrix, and ***G***(***x***) is a 10 × 10 function matrix. They can be expressed in concrete formulas as:

(21) $$\begin{array}{l} F_{c}(x)=\\ \left(\begin{array}{l} {\tilde{f}}_{ibz}^{b}\;(\cos\psi \;\sin\gamma \;+\;\cos\gamma \sin\psi \;\sin\theta )\;-\;(\cos\psi \;\cos\gamma \;-\;\sin\psi \;\sin\theta \;\sin\gamma )\;(\nabla_{ax}\;-\;{\tilde{f}}_{ibx}^{b})\;+v_{north}(2\omega_{ie}\sin L+\frac{v_{east}\tan L}{r_{N}})+\cos\theta \sin\psi (\nabla_{ay}-{\tilde{f}}_{iby}^{b})\\ {\tilde{f}}_{ibz}^{b}\;(\sin\psi \;\sin\gamma \;-\;\cos\psi \cos\gamma \;\sin\theta )\;-\;(\;\cos\gamma \;\sin\psi +\;\cos\psi \;\sin\theta \;\sin\gamma )\;(\nabla_{ax}\;-\;{\tilde{f}}_{ibx}^{b})\;-v_{east}(2\omega_{ie}\sin L+\frac{v_{east}\tan L}{r_{N}})-\cos\psi \cos\theta (\nabla_{ay}-{\tilde{f}}_{iby}^{b})\\ {\tilde{\omega }}_{ibx}^{b}\cos\gamma \;+\;{\tilde{\omega }}_{ibz}^{b}\sin\gamma \;-\;\varepsilon_{gx}\cos\gamma \;-\;\varepsilon_{gz}\sin\gamma \;+\;\frac{v_{north}\cos\psi }{r_{M}}-\;\omega_{ie}\cos L\sin\psi \;-\frac{v_{east}}{r_{N}}\sin\psi \\ {\tilde{\omega }}_{iby}^{b}\;-\;\varepsilon_{gy}\;-\;{\tilde{\omega }}_{ibz}^{b}\cos\gamma \tan\theta \;+\;{\tilde{\omega }}_{ibx}^{b}\sin\gamma \tan\theta \;+\;\varepsilon_{gz}\cos\gamma \tan\theta \;-\;\varepsilon_{gx}\sin\gamma \tan\theta \;-\;\frac{v_{north}\sin\psi }{r_{M}\cos\theta }\;-\;\omega_{ie}\cos L\frac{\cos\psi }{\cos\theta }\;-\;\frac{v_{east}\cos\psi }{r_{N}\cos\theta }\\ \frac{{\tilde{\omega }}_{ibz}^{b}\cos\gamma }{\cos\theta }-\;\frac{v_{east}}{r_{N}}\tan L\;-\;\omega_{ie}\sin L\;-\;\frac{{\tilde{\omega }}_{ibx}^{b}\sin\gamma }{\cos\theta }\;-\;\frac{\varepsilon_{gz}\cos\gamma }{\cos\theta }+\frac{\varepsilon_{gx}\sin\gamma }{\cos\theta }\;+\;\frac{v_{north}}{r_{M}}\sin\psi \tan\theta \;+\;\omega_{ie}\cos L\cos\psi \tan\theta \;+\frac{v_{east}}{r_{N}}\cos\psi \tan\theta \\ 0_{5\times 1} \end{array}\right) \end{array}$$

(22) $$G(x)=\left[\begin{array}{c} \begin{array}{cc} G_{1}(x) & 0_{2\times 3}\\ 0_{3\times 2} & G_{2}(x) \end{array}\;0_{5\times 5}\\ \cdots \cdots \cdots \cdots \cdots \cdots \cdots \cdots \\ \begin{array}{cc} & 0_{5\times 10} \end{array} \end{array}\right]$$

(23) $$G_{1}(x)=\left[\begin{array}{cc} \cos\psi \cos\gamma \;-\;\sin\psi \sin\theta \sin\gamma & -\cos\theta \sin\psi \\ \cos\gamma \sin\psi \;+\;\cos\psi \sin\theta \sin\gamma & \cos\psi \cos\theta \end{array}\right]$$

(24) $$G_{2}(x)=\left[\begin{array}{ccc} \cos\gamma & 0 & \sin\gamma \\ \tan\theta \sin\gamma & 1 & -\;\cos\gamma \tan\theta \\ -\frac{\sin\gamma }{\cos\theta } & 0 & \frac{\cos\gamma }{\cos\theta } \end{array}\right]$$

where **0***_i × j_* denotes a *i* × *j* zero matrix.

Equation (20) is the continuous nonlinear system equation. A fourth-order Runge-Kutta scheme with two steps between successive inertial sensor sampling instances is employed for numerical integration $\dot{x}=F_{c}(x)$. Then Equation (20) is assumed to be converted to a difference equation shown as follows:

(25) $$x_{k}=F(x_{k-1})+w_{k-1}$$

where ***x_k_*** and ***x_k_*_− 1_** represent the state ***x*** at time *t_k_* and *t_k_*_− 1_ respectively, ***F*** (***x_k_*_− 1_**) represents the discrete nonlinear state transfer function, which can be supposed to be the integration form of ***F_c_***(***x***) at the state ***x_k_*** **_− 1_** within the integration period denoted as the time interval *T*_1_, ***w_k_*_− 1_** represents the discrete system noise at time *t**_k_*** **_− 1_**, $E[w_{k}]=0,E[w_{k}w_{j}^{T}]=Q_{k}\delta_{kj}$, $Q_{k}=G(x_{k})QG{\left(x_{k}\right)}^{T}T_{1}$, where ***w****_k_* and ***w****_j_* represent the discrete system noise at time *t_k_* and *t_j_*, respectively, and ***w****_k_* will be characterized by the covariance matrice ***Q****_k_*, *δ_kj_* is the Dirichlet function which means that *δ_kj_* is equal to 1 when *k* is equal to *j,* and otherwise *δ_kj_* is equal to 0, and ***G***(***x****_k_*) is the value of ***G***(***x***) at time *t_k_*.

### 2.2. Measurement Model

We directly choose the *b*-frame velocity from sensor outputs as the filter measurement, which can be expressed as:

(26) $$z={\left[{\tilde{v}}_{bx},{\tilde{v}}_{by},{\tilde{v}}_{bz}\right]}^{T}$$

where ***z*** is the three-dimensional filter measurement vector.

The *b*-frame velocity measurements are compared with the corresponding quantities from the SINS. Estimates of the *b*-frame velocity may be obtained from the SINS estimates of the *n*-frame velocity (*v_east_*, *v_north_*, *v_up_*) and the $C_{n}^{b}$, therefore, the filter measurement equation can be expressed as:

(27) $$z=C_{n}^{b}{\left[v_{east},v_{north},v_{up}\right]}^{T}+\eta$$

(28) $$\eta ={\left[\eta_{vbx},\eta_{vby},\eta_{vbz}\right]}^{T}$$

where ***η*** is the three-dimensional filter measurement noise vector that accounts for the error in the aid sensor measurement, *η_vbx_*, *η_vby_* and *η_vbz_* denote the measurement noise components along *x*, *y*, *z*-axis of body. ***η*** is assumed to be a zero mean and additive white Gaussian noise with a power spectral density of ***R***. ***R*** is a 3 × 3 diagonal matrix, the elements of which are selected in accordance with the anticipated level of velocity measurement noise.

According to the filter measurement in Equation (26), the filter measurement noise vector in Equation (28) and the state vector in Equation (18), Equation (27) is written in state space form as:

(29) z=H(x)+η

where ***H***(***x***) is the nonlinear measurement function, which is derived from Equation (27). ***H***(***x***) is a 3 × 1 function matrix as follows:

(30) $$H(x)=\left[\begin{array}{l} v_{east}(\cos\psi \cos\gamma \;-\;\sin\psi \sin\theta \sin\gamma )\;+v_{north}(\cos\gamma \sin\psi \;+\;\cos\psi \sin\theta \sin\gamma )\\ v_{north}\cos\psi \cos\theta \;-\;v_{east}\cos\theta \sin\psi \\ v_{east}(\cos\psi \sin\gamma \;+\;\cos\gamma \sin\psi \sin\theta )\;+\;v_{north}(\sin\psi \sin\gamma \;-\;\cos\psi \cos\gamma \sin\theta ) \end{array}\right]$$

The measurement equation at time *t_k_* expressed in terms of the state can be shown as follows:

(31) $$z_{k}=H(x_{k})+\eta_{k}$$

where ***z****_k_* is the filter measurement at time *t_k_*, ***H***(***x****_k_*) is the value of ***H***(***x***) at time *t_k_*, ***η****_k_* is the discrete filter measurement noise vector at time *t_k_*, and ***η****_k_* is a zero mean white noise sequence that can be characterized by the covariance matrice ***R****_k_* as follows:

(32) $$E[\eta_{k}]=0,E[\eta_{k}\eta_{j}^{T}]=R_{k}\delta_{kj},R_{k}=R/T_{2}$$

where *T*_2_ is the aid sensor data updating period, and ***η****_j_* is the discrete filter measurement noise vector at time *t_j_*. The terms ***w****_k_* and ***η****_k_* are assumed to be independent, i.e.,:

(33) $$E[w_{k}\eta_{j}^{T}]=0$$

### 2.3. Filter State Model Analysis

The system model in Equation (25) and the measurement model in Equation (31) construct the filter state model. For the purpose of illustration, the state vector of conventional integrated navigation model in many references usually consists of three attitude errors, three velocity errors, three position errors, and the inertial sensor errors. Well-known SINS error models are derived from the perturbation theory. After the errors are estimated, the navigation information calculated by the SINS navigation algorithm is corrected by these errors.

In this paper, navigation parameters instead of their associated errors are chosen as the states. The system model in Equation (25), which contains the differential equations of velocity, Euler angle, and inertial sensor bias, is nonlinear and simple without any restrictions and approximations. The system model is also the SINS mechanization. Note that the system equations are considered a direct formation, as opposed to the alternative indirect (error) formulation. The navigation information is obtained from the states directly after the filtering, so the error correction is no longer needed. The navigation computation is performed simultaneously in the filter time updating.

For the *b*-frame velocity aided SINS like SINS/DVL and SINS/Odometer in the indirect approaches aforementioned, the velocity measured in the *b*-frame is usually first transformed into the velocity expressed in the *n*-frame using the DCM calculated by the SINS. Then the difference of the transformed velocity and the corresponding quantity from the SINS is used as the filter measurement. The measurement models are indirect and linear. The cross-noise will arise from the formation of the filter measurement [6].

In this research, the aid outputs are chosen as shown in Equation (26), instead of the associated difference, as the filter measurements. The measurement equations contain the aid measurement noise, while the system equations contain the inertial sensor noise; therefore, the inertial sensor noise is separated from the aid measurement noise. The cross-noise problem is solved. The measurement model is direct and nonlinear. The velocity transformation is realized in the measurement equations. Hence the measurement model in Equation (31) is more accurate and easier to be comprehended, compared with those in the indirect approaches.

In Section 2, the system equations and the measurement equations are developed. An UKF will be employed to estimate the state vector because of their high nonlinearity. In the succeeding section (Section 3), the UKF is presented in detail.

## 3. UKF

The UKF uses the unscented transformation (UT) which is developed as a method to propagate mean and covariance information through nonlinear transformations. The UT is founded on the intuition that it is easier to approximate a probability distribution than to approximate an arbitrary nonlinear function or transformation. The UT chooses a set of sigma points to approximate a Gaussian distribution. The sigma points match the mean and covariance. The nonlinear function is applied to each point, and in turn yields a cloud of transformed points. The statistics of the transformed points can then be calculated to form an estimate of the nonlinearly transformed mean and covariance. A description of the UKF can be found in [9,13,14].

We summarize the steps involved in the time update and the measurement update of an UKF as shown in Algorithm 1.

**Algorithm 1.** UKF  **1. Initialize**
(34)${\hat{x}}_{0}=E(x_{0}),P_{0}=E(({\hat{x}}_{0}-x_{0}){\left({\hat{x}}_{0}-x_{0}\right)}^{T})$where ***x***_0_ and ***P***_0_ are the initial state and the initial covariance matrix, respectively.  **2. Time update**  Factorize
(35)$P_{k-1|k-1}=S_{k-1|k-1}S_{k-1|k-1}^{T}$  Evaluate the sigma points (*j* = 1, 2… *N_s_*):
(36)$\chi_{j,k-1|k-1}=S_{k-1|k-1}\xi_{j}+{\hat{x}}_{k-1|k-1}$  Evaluate the propagated sigma points (
*j* = 1, 2… *N_s_*):
(37)$\chi_{j,k|k-1}^{*}=F(\chi_{j,k-1|k-1})$  Estimate the predicted state:
(38)${\hat{x}}_{k|k-1}=\sum_{j=1}^{N_{s}}\chi_{j,k|k-1}^{*}a_{j}$  Estimate the predicted state error covariance:
(39)$P_{k|k-1}=\sum_{j=1}^{N_{s}}a_{j}\chi_{j,k|k-1}^{*}\chi_{j,k|k-1}^{*T}-{\hat{x}}_{k|k-1}{\hat{x}}_{k|k-1}^{T}+Q_{k-1}$where ***ξ**_j_* and *a_j_* are the *j*-th sigma point and the associated weight, respectively, *N_s_* denotes the number of the sigma points, and ***Q**_k_*_-1_ is the discrete system noise covariance matrix at time *t_k_*_− 1_.  **3. Measurement update**  Factorize:
(40)$P_{k|k-1}=S_{k|k-1}S_{k|k-1}^{T}$  Evaluate the sigma points (
*j* = 1, 2… *N_s_*):
(41)$\chi_{j,k|k-1}=S_{k|k-1}\xi_{j}+{\hat{x}}_{k|k-1}$  Evaluate the propagated sigma points (*j* = 1, 2… *N_s_*):
(42)$Z_{j,k|k-1}=H(\chi_{j,k|k-1})$  Estimate the predicted measurement:
(43)${\hat{z}}_{k|k-1}=\sum_{j=1}^{N_{s}}Z_{j,k|k-1}a_{j}$  Estimate the innovation covariance matrix:
(44)$P_{zz,k|k-1}=\sum_{j=1}^{N_{s}}a_{j}Z_{j,k|k-1}Z_{j,k|k-1}^{T}-{\hat{z}}_{k|k-1}{\hat{z}}_{k|k-1}^{T}+R_{k}$  Estimate the cross-covariance matrix:
(45)$P_{xz,k|k-1}=\sum_{j=1}^{N_{s}}a_{j}\chi_{j,k|k-1}Z_{j,k|k-1}^{T}-{\hat{x}}_{k|k-1}{\hat{z}}_{k|k-1}^{T}$  Estimate the Kalman gain:
(46)$K_{k}=P_{xz,k|k-1}P_{zz,k|k-1}^{-1}$  Estimate the updated state:
(47)${\hat{x}}_{k|k}={\hat{x}}_{k|k-1}+K_{k}(z_{k}-{\hat{z}}_{k|k-1})$  Estimate the corresponding error covariance: 
(48)$$P_{k|k}=P_{k|k-1}-K_{k}P_{zz,k|k-1}K_{k}^{T}$$

There are a series of techniques to determine the sigma points [14]. The symmetric sigma point sets are selected, which is the most commonly used UT [13]. The sigma points are given by:

(49) $$\xi_{j}=\{\begin{array}{c} {\left[0,0,\cdots 0\right]}^{T},j=1\\ (\sqrt{d+\kappa }){\left[1\right]}_{j},j=2,3,\cdots N_{s} \end{array}$$

(50) $${\left[1\right]}_{j}\in \left\{\left(\begin{array}{c} 1\\ 0\\ \vdots \\ 0 \end{array}\right),\left(\begin{array}{c} 0\\ 1\\ \vdots \\ 0 \end{array}\right),\cdots ,\left(\begin{array}{c} 0\\ 0\\ \vdots \\ 1 \end{array}\right),\left(\begin{array}{c} -1\\ 0\\ \vdots \\ 0 \end{array}\right),\left(\begin{array}{c} 0\\ -1\\ \vdots \\ 0 \end{array}\right),\cdots ,\left(\begin{array}{c} 0\\ 0\\ \vdots \\ -1 \end{array}\right)\right\}$$

(51) $$a_{j}=\left\{ \begin{array}{l} \frac{\kappa }{d+\kappa },j=1\\ \frac{1}{2(d+\kappa )},j=2,3,\cdots N_{s} \end{array}\right.$$

*N_s_* = 2*d* + 1 (52)

where *d* is the dimension of the states and *d* is equal to 10 here as shown in Equation (18), *κ* = −7 is chosen in accordance with the heuristic *d* + *κ* = 3, [1]*_j_* is the *j*-th column vector, the right-hand side of (50) has 2*d* column vectors, each of which has *d* components.

Note that the period of the time updating differs from that of the measurement updating. Since the system equations include the inertial sensor outputs, the time updating is processed once new data of the inertial sensors arrive, and thus the time updating period is *T*_1_. The time updating is implemented at a fast inertial sensor sample rate, e.g., 200 Hz here. At the end of the time updating, if the aid measurements do not arrive, the predicted state ${\hat{x}}_{k|k-1}$ and the predicted error covariance $P_{k|k-1}$ are transferred to the updated state ${\hat{x}}_{k|k}$ and the updated error covariance $P_{k|k}$, respectively, the first step of time updating is then repeated, and the new time updating loop begins. Upon the arrival of a new navigation aid measurement, the measurement updating is processed, and thus the measurement period is *T*_2_. The measurement updating is implemented at a slow navigation aid sample rate, e.g., 10 Hz here.

## 4. Experimental Results

The purpose of the experiment is to show the advantages of the new direct algorithm over the typical indirect Kalman filter.

**Figure 2:**
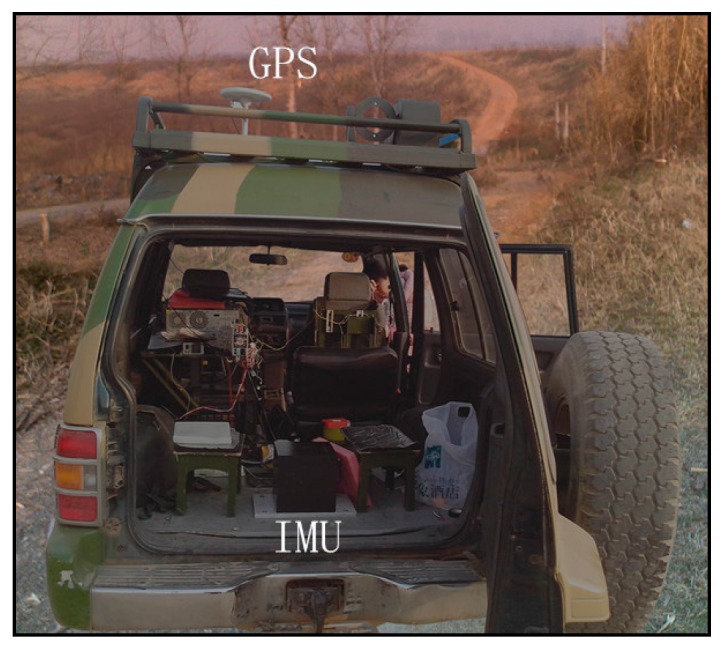
Test vehicle.

The performance of the proposed direct UKF approach for the aided SINS is examined with road test data from a land vehicle. The test vehicle is shown in Figure 2. The SINS/odometer integrated navigation system is used. The navigation aid is a precision odometer from which the forward speed is obtained. The odometer is installed at the right rear wheel. The inertial sensors used in the experiments are from a navigation grade IMU. A GPS is installed on the top of the vehicle. Note that the GPS is not integrated in the navigation system, but it is only used as the position reference. The GPS receiver provides position with precision of about 10 m, and update rates up to 1 Hz.

The IMU contains three fiber optic gyros and three quartz accelerometers. The inertial sensor data are sampled at 200 Hz (*T*_1_ = 5 ms). The fixed biases for the gyros and accelerometers used here are 0.03°/h (1 σ) and 0.2 mg (1 σ), respectively.

The odometer only provides the forward speed with precision of about 0.1 m/s at the sample rate 10 Hz (*T*_2_ = 0.1 s). Note that the nonholonomic constraints on the land vehicle arise from the fact that the vehicle cannot move in the transversal or the vertical directions in the *b*-frame. In addition, the IMU-odometer misalignment angle and odometer scale factor are calibrated prior to the field test. It can be assumed that the odometer measurement error is an additional white Gauss noise. Hence, the measurement of the UKF is given by:

$$z={\left[\begin{array}{ccc} 0, & {\tilde{v}}_{by}, & 0 \end{array}\right]}^{T}$$

where ${\tilde{v}}_{by}$ is the forward speed derived from the odometer.

The initial state vector of the filter is set as:

$$x_{0}={\left[\begin{array}{cccccccccc} -{\tilde{v}}_{by0}\sin\left(\psi_{0}\right)\cos\left(\theta_{0}\right), & {\tilde{v}}_{by0}\cos\left(\psi_{0}\right)\cos\left(\theta_{0}\right), & \theta_{0}, & \gamma_{0}, & \psi_{0}, & 0, & 0, & 0, & 0, & 0 \end{array}\right]}^{T}$$

where ${\tilde{v}}_{by0}$ is the initial forward speed from the odometer, and *θ*_0_, *γ*_0_, *ψ*_0_ are the initial Euler angles that are transferred to the filter at the end of the alignment. Apparently, unlike the conventional approaches that use the errors as the states, ***x*_0_** is not zero here because it stands for the initial navigation information which is set according to the results of the initial alignment. Given the relationship between the vehicle velocities in the *b*-frame and in the *n*-frame, the first two components of ***x*_0_** are calculated by the DCM derived from the alignment results.

The other initial parameters of the filter are set as:

***P***_0_ = diag{(0.1 m/s)^2^, (0.1 m/s)^2^, (0.1°)^2^, (0.1°)^2^, (0.3°)^2^, (0.2 mg)^2^, (0.2 mg)^2^, (0.03°/h)^2^, (0.03°/h)^2^, (0.03°/h)^2^}
***Q*** = diag{(0.2 mg)^2^, (0.2 mg)^2^, (0.03°/h)^2^, (0.03°/h)^2^, 0.03°/h)^2^,0,0,0,0,0}
***R*** = diag{(0. 1 m/s)^2^, (0.1 m/s)^2^, (0.1 m/s)^2^}

where diag{·} denotes the diagonal matrix. ***P***_0_ is set according to the accuracy of the alignment and the inertial sensor biases, ***Q*** is set according to the inertial sensor noises, and ***R*** is set according to the odometer noises. They can be slightly tuned to make the filter converge rapidly.

After the vehicle engine was started, the vehicle was stationary at the initial position to process the initial alignment during the first five minutes. Then the vehicle ran along the trajectory until it reached the end. Three road test trajectories were carried out. The three trajectory information (time and distance) are shown in Table 1, where the time includes the five minutes for the alignment.

The experiment results of the proposed direct UKF solution for SINS/odometer integration (denoted as Direct) is compared with the standard indirect approach utilizing the KF and SINS error models introduced in Section 1 (denoted as Indirect). The position errors of the direct and indirect solutions are calculated with respect to the longitude and latitude provided by GPS.

**Figure 3:**
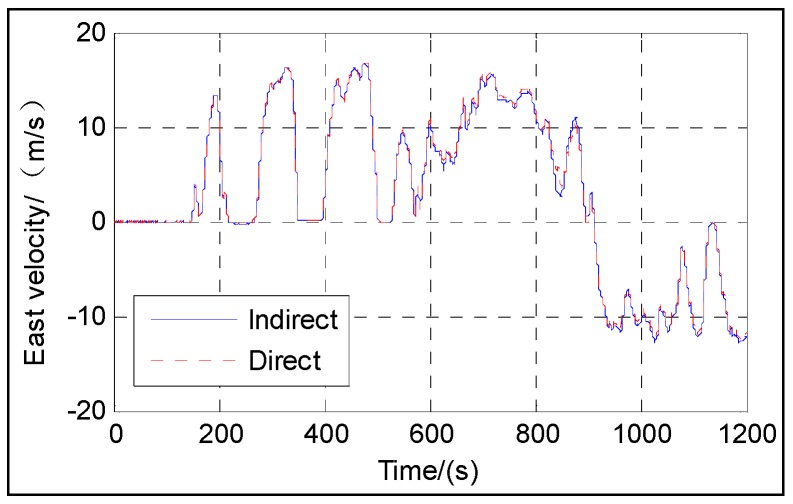
East velocity.

**Figure 4:**
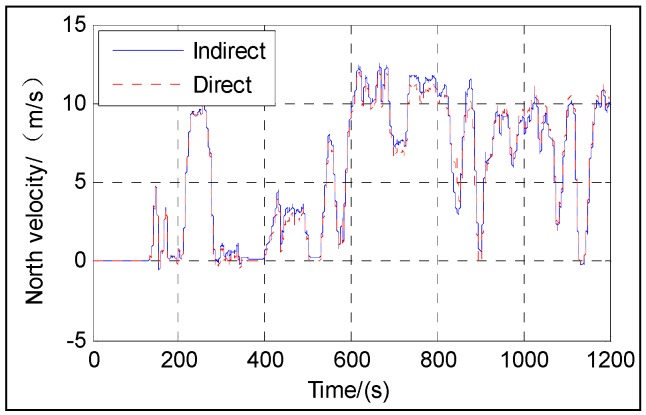
North velocity.

**Figure 5:**
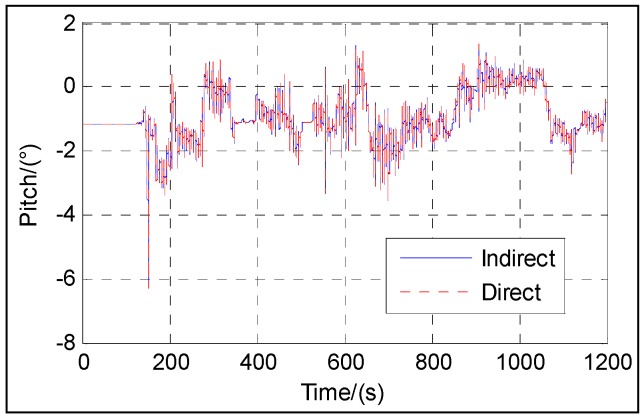
Pitch.

**Figure 6:**
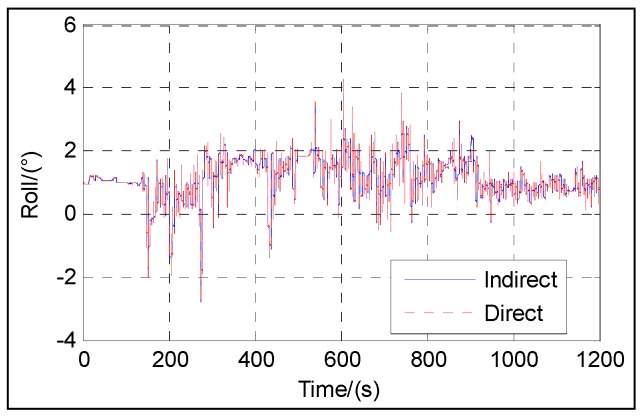
Roll.

**Figure 7:**
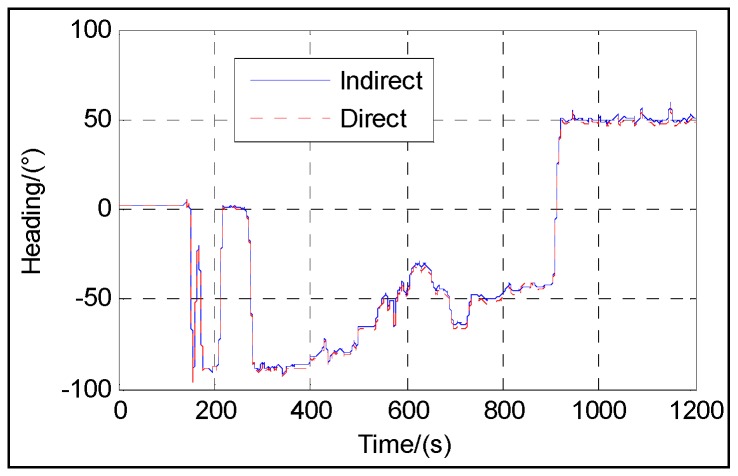
Heading.

Figure 3, Figure 4, Figure 5, Figure 6 and Figure 7 show the velocity and attitude of the SINS/odometer integrated navigation system in the integrated navigation phase of about 20 min during Trajectory 1. The velocity and attitude are directly estimated by the UKF in the direct approach, while in indirect approach, the velocity and attitude are calculated by the SINS algorithm first and then corrected, employing the errors estimated by the KF. *v_east_* and *v_north_* are shown in Figure 3 and Figure 4, respectively, and Figure 5, Figure 6 and Figure 7 show the pitch, roll and heading, respectively.

From Figure 3, Figure 4, Figure 5, Figure 6 and Figure 7, we can see that the proposed direct approach can achieve the velocity and attitude similar to the indirect approach, and thus it can also capture the vehicle motion.

**Figure 8:**
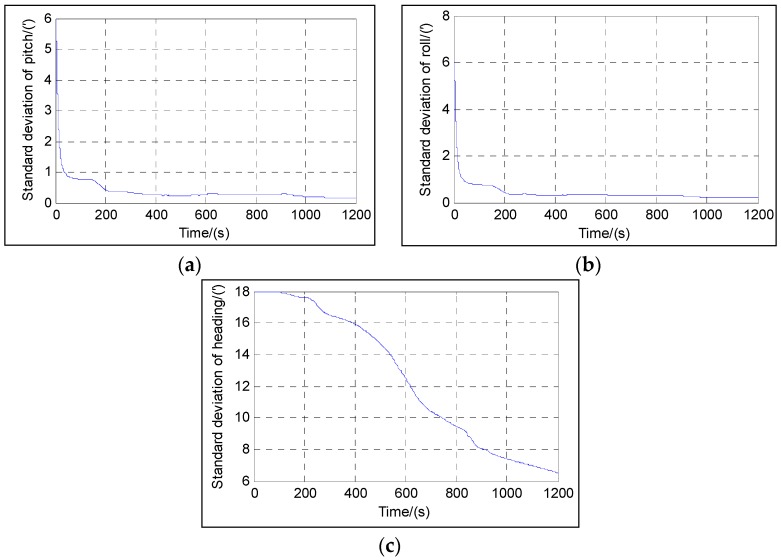
Attitude standard deviation of the direct approach: (**a**) Standard deviation of pitch; (**b**) Standard deviation of roll; (**c**) Standard deviation of heading.

As the states of the direct approach differ from those of the indirect approach, we use the standard deviations of the attitude and the attitude errors to illustrate the convergence trend in the direct and indirect approaches, respectively. Comparing the standard deviations of the attitude with the attitude error is meaningless, as they are different quantities. Moreover, the indirect approach has been well-studied. Here we only show the standard deviations of the attitude in the direct approach during Trajectory 1 as in Figure 8.

From Figure 8, it can be seen that the attitude standard deviations all decrease when time passes. Figure 8a,b shows that the pitch and roll converge very rapidly as the system effectively aligns itself to the local gravity vector, and they converge at about 20 s, and their standard deviations settle to less than 0.1′, and their accuracy is limited by any residual bias in the accelerometer measurements. Figure 8c shows that the heading converges slowly due to its lower observability as the heading error only propagates as a velocity error and therefore becomes more observable when the vehicle maneuvers, and its standard deviation settles to about 6.5′ at the end, and the accuracy is mainly limited by any residual bias in the gyro measurements. The attitude convergence characteristics show that not only attitude estimates can be achieved using the *b*-frame velocity as the filter measurement, but they also conform to the observability analysis.

**Figure 9:**
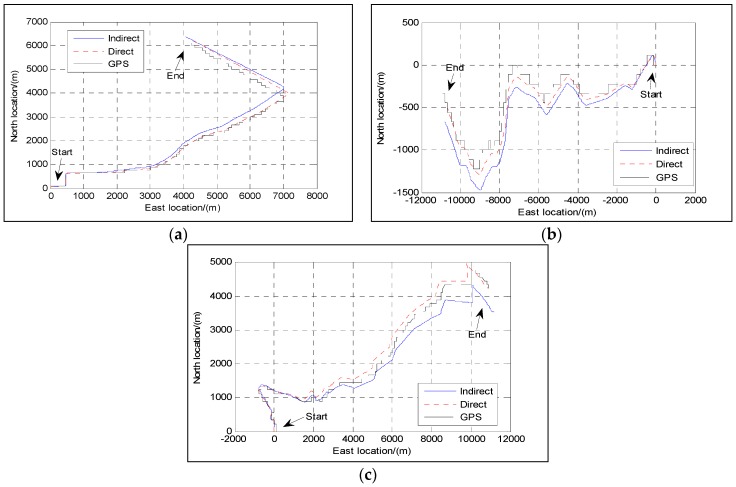
Position: (**a**) Trajectory 1; (**b**) Trajectory 2; (**c**) Trajectory 3.

Figure 9 shows the position information of the GPS as the reference, the position information of the indirect approach and the position information of the proposed direct approach for the SINS/Odometer integrated navigation system during three different trajectories. In Figure 9, the longitude and the latitude are converted into the east location and the north location in meters (not degrees) for clarity.

From Figure 9, it can be seen obviously that the position errors increase with the time during different trajectories in both approaches. Although the velocity errors are bounded with the velocity aid, the remainder velocity errors that accumulate in the integration process result in the increase of position errors. Compared with the indirect approach, it is also clear that the direct approach makes it possible to obtain position curves that are closer to those of the GPS. The position errors defined by the Euclidian distance between the estimated position and the GPS position at the end of the trajectories are shown in Table 2 for both approaches.

In Table 2, the improvement on navigation performance of the direct approach is shown clearly, and its position errors are much smaller than those of the indirect approach. Position errors are related to the accuracy of the velocity and attitude estimates. The models developed in Section 2 in the direct approach almost involve no approximation, and the linearization and the cross-noise problems are avoided. They are more accurate and can capture the SINS nonlinearity better than the SINS error models. Moreover, the nonlinear models enable the UKF to estimate the navigation parameters directly, and the UKF shows better performance than the KF in such nonlinear cases.

## 5. Singularity Problem

Equation (12) is singular when *θ* = ±π/2. In this section, the dual-Euler method is adopted to remove the singularities at *θ* = ±π/2. The 3-2-1 and 3-1-2 Euler angle sets are used. If *θ* is close to ±π/2, the 3-2-1 Euler angle set is singular while the Euler angle set 3-1-2 is non-singular [29]. We can switch between these two sets when *θ* is close to ±π/4.

The heading, pitch and roll of the 3-1-2 Euler angle set are denoted as *ψ_r_*, *θ_r_*, *γ_r_* respectively with the subscript *r* to show the difference from the 3-2-1 Euler angle set. The three separate direction cosine matrices are defined as $C_{\psi_{r}}$, $C_{\gamma_{r}}$ and $C_{\theta_{r}}$, respectively. Thus, a transformation from reference to body axes, denoted as $C_{rn}^{b}$, may be expressed as the product of these three separate transformations as follows:

(53) $$\begin{array}{l} C_{rn}^{b}=C_{\theta_{r}}C_{\gamma_{r}}C_{\psi_{r}}=\left[\begin{array}{ccc} 1 & 0 & 0\\ 0 & \cos\theta_{r} & \sin\theta_{r}\\ 0 & -\sin\theta_{r} & \cos\theta_{r} \end{array}\right]\left[\begin{array}{ccc} \cos\gamma_{r} & 0 & -\sin\gamma_{r}\\ 0 & 1 & 0\\ \sin\gamma_{r} & 0 & \cos\gamma_{r} \end{array}\right]\left[\begin{array}{ccc} \cos\psi_{r} & \sin\psi_{r} & 0\\ -\sin\psi_{r} & \cos\psi_{r} & 0\\ 0 & 0 & 1 \end{array}\right]\\ =\left[\begin{array}{ccc} \cos\gamma_{r}\cos\psi_{r} & \cos\gamma_{r}\sin\psi_{r} & -\sin\gamma_{r}\\ \cos\psi_{r}\sin\gamma_{r}\sin\theta_{r}-\;\cos\theta_{r}\sin\psi_{r} & \cos\psi_{r}\cos\theta_{r}\;+\;\sin\psi_{r}\sin\gamma_{r}\sin\theta_{r} & \cos\gamma_{r}\sin\theta_{r}\\ \sin\psi_{r}\sin\theta_{r}\;+\;\cos\psi_{r}\cos\theta_{r}\sin\gamma_{r} & \cos\theta_{r}\sin\psi_{r}\sin\gamma_{r}\;-\;\cos\psi_{r}\sin\theta_{r} & \cos\gamma_{r}\cos\theta_{r} \end{array}\right] \end{array}$$

Similarly, Equations (12) should be changed for the 3-1-2 Euler angle set as follows:

(54) $$\left[\begin{array}{c} {\dot{\theta }}_{r}\\ {\dot{\gamma }}_{r}\\ {\dot{\psi }}_{r} \end{array}\right]=\frac{1}{\cos\gamma_{r}}\left[\begin{array}{ccc} \cos\gamma_{r} & \sin\gamma_{r}\sin\theta_{r} & \sin\gamma_{r}\cos\theta \\ 0 & \cos\theta_{r}\cos\gamma_{r} & -\sin\theta_{r}\cos\gamma_{r}\\ 0 & \sin\theta_{r} & \cos\theta_{r} \end{array}\right]\omega_{nb}^{b}$$

where $\omega_{nb}^{b}$ is given by:

(55) $$\omega_{nb}^{b}=\omega_{ib}^{b}-C_{rn}^{b}(\omega_{ie}^{n}+\omega_{en}^{n})$$

The solutions of the ${\dot{\theta }}_{r}$ and ${\dot{\psi }}_{r}$ equations become indeterminate when $\gamma_{r}$ = ±π/2. $\gamma_{r}$ = ±π/2 are the singularities of the 3-1-2 Euler angle set.

The 3-2-1 and 3-1-2 Euler angle sets show difference in the rotation sequence. However, both $C_{rn}^{b}$ and $C_{n}^{b}$ represent the transformation from reference to body axes, if:

$$C_{n}^{b}=C_{rn}^{b}=\left[\begin{array}{ccc} c_{11} & c_{12} & c_{13}\\ c_{21} & c_{22} & c_{23}\\ c_{31} & c_{32} & c_{33} \end{array}\right]$$

Combining Equation (12) with Equation (53), the equation given above can be written as:

(56) $$\begin{array}{l} \left[\begin{array}{ccc} c_{11} & c_{12} & c_{13}\\ c_{21} & c_{22} & c_{23}\\ c_{31} & c_{32} & c_{33} \end{array}\right]\\ =\left[\begin{array}{ccc} \cos\gamma \cos\psi -\sin\theta \sin\gamma \sin\psi & \cos\gamma \sin\psi +\sin\theta \sin\gamma \cos\psi & -\cos\theta \sin\gamma \\ -\cos\theta \sin\psi & \cos\theta \cos\psi & \sin\theta \\ \sin\gamma \cos\psi +\sin\theta \cos\gamma \sin\psi & \sin\gamma \sin\psi -\sin\theta \cos\gamma \cos\psi & \cos\theta \cos\gamma \end{array}\right]\\ =\left[\begin{array}{ccc} \cos\gamma_{r}\cos\psi_{r} & \cos\gamma_{r}\sin\psi_{r} & -\sin\gamma_{r}\\ \cos\psi_{r}\sin\gamma_{r}\sin\theta_{r}-\;\cos\theta_{r}\sin\psi_{r} & \cos\psi_{r}\cos\theta_{r}\;+\;\sin\psi_{r}\sin\gamma_{r}\sin\theta_{r} & \cos\gamma_{r}\sin\theta_{r}\\ \sin\psi_{r}\sin\theta_{r}\;+\;\cos\psi_{r}\cos\theta_{r}\sin\gamma_{r} & \cos\theta_{r}\sin\psi_{r}\sin\gamma_{r}\;-\;\cos\psi_{r}\sin\theta_{r} & \cos\gamma_{r}\cos\theta_{r} \end{array}\right] \end{array}$$

Hence, the relationship between the Euler angles of the 3-2-1 and 3-1-2 Euler angle sets can be expressed by:

(57) $$\left[\begin{array}{c} \theta_{r}\\ \gamma_{r}\\ \psi_{r} \end{array}\right]=\left[\begin{array}{c} \mathrm{arctg}(\frac{c_{23}}{c_{33}})\\ -\arcsin c_{13}\\ \mathrm{arct}g(\frac{c_{12}}{c_{11}}) \end{array}\right]=\left[\begin{array}{c} \mathrm{arctg}(\frac{\sin\theta }{\cos\theta \cos\gamma })\\ -\arcsin(-\cos\theta \sin\gamma )\\ \mathrm{arct}g(\frac{\cos\gamma \sin\psi +\sin\theta \sin\gamma \cos\psi }{\cos\gamma \cos\psi -\sin\theta \sin\gamma \sin\psi }) \end{array}\right]\left(\gamma_{r}\neq \pm \pi /2\right)$$

and:

(58) $$\left[\begin{array}{c} \theta \\ \gamma \\ \psi \end{array}\right]=\left[\begin{array}{c} \arcsin c_{23}\\ \mathrm{arct}g(-\frac{c_{13}}{c_{33}})\\ \mathrm{arct}g(-\frac{c_{21}}{c_{22}}) \end{array}\right]=\left[\begin{array}{c} \arcsin(\cos\gamma_{r}\sin\theta_{r})\\ \mathrm{arct}g(\frac{\sin\gamma_{r}}{\cos\gamma_{r}\cos\theta_{r}})\\ \mathrm{arct}g(-\frac{\cos\psi_{r}\sin\gamma_{r}\sin\theta_{r}-\;\cos\theta_{r}\sin\psi_{r}}{\cos\psi_{r}\cos\theta_{r}\;+\;\sin\psi_{r}\sin\gamma_{r}\sin\theta_{r}}) \end{array}\right]\left(\theta \neq \pm \pi /2\right)$$

Substituting with *θ* = ±π/2 in Equation (56) yields $c_{13}=-\sin\gamma_{r}=-\cos\theta \sin\gamma =0$, and then *γ_r_* = 0 or π and *θ_r_* = ±π/2 are obtained. Substituting with *γ_r_* = ±π/2 in Equation (56) yields $c_{23}=\cos\gamma_{r}\sin\theta_{r}=\sin\theta =0$, and then *θ* = 0 or π and *γ* = ±π/2 are obtained. Therefore, the singularities of the 3-2-1 and 3-1-2 Euler angle sets are complementary.

The differences between the system model and measurement model of the direct approach using the 3-1-2 and 3-2-1 Euler angle sets are the DCM and the Euler angles. Substituting *ψ_r_*, *θ_r_*, *γ_r_* for *θ*, *γ*, *ψ* in Equation (18), respectively, yields the state:

(59) $$x_{r}={\left[v_{east},v_{north},\theta_{r},\gamma_{r},\psi_{r},\nabla_{ax},\nabla_{ay},\varepsilon_{gx},\varepsilon_{gy},\varepsilon_{gz}\right]}^{T}$$

The navigation equation as shown in Equation (1) is changed by substituting $C_{rn}^{b}$ for $C_{n}^{b}$ in Equation (3). Then it is combined with Equation (54) and Equation (17), and the system model of the direct approach using the 3-1-2 Euler angle set is yielded. The corresponding filter measurement equation is yielded by substituting $C_{rn}^{b}$ for $C_{n}^{b}$ in Equation (27).

**Figure 10:**
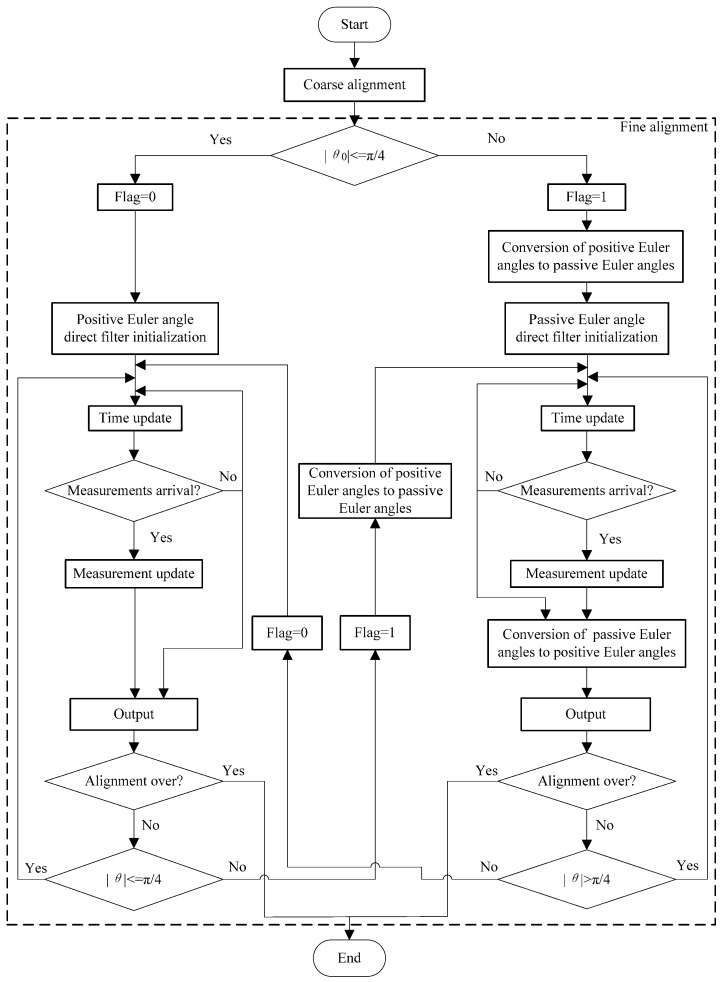
Flow chart of the SINS initial alignment using the dual-Euler direct approach.

The flow chart of the direct fusing approach with the dual-Euler method, taking the SINS initial alignment for example, is illustrated in Figure 10. For the purpose of simplicity, the direct approach using the 3-2-1 Euler angle set is denoted as the positive Euler angle direct approach, and the direct approach using the 3-1-2 Euler angle set is denoted as the passive Euler angle direct approach, and correspondingly, the Euler angles are denoted as the positive Euler angles and the passive Euler angles, respectively. When flag = 0, the positive Euler angle direct approach works; when flag = 1, the passive Euler angle direct approach works.

As shown in Figure 10, the two branches are almost the same except for two steps: for passive Euler angle direct approach, positive Euler angles are converted to passive Euler angles at the beginning of the filtering, and passive Euler angles are converted to positive Euler angles at the end of the filtering, since the outputs are expressed in terms of positive Euler angles. When the switching from the positive Euler angle direct branch to the passive Euler angle direct branch occurs, the positive Euler angles in the filter state is converted to the passive Euler angles according to Equation (57), and then they are transferred to the filter state of the passive Euler angle direct branch. Notably, it is unnecessary to change the other parameters of the filter. Equation (58) is used for the conversion in the inverse switch.

**Figure 11:**
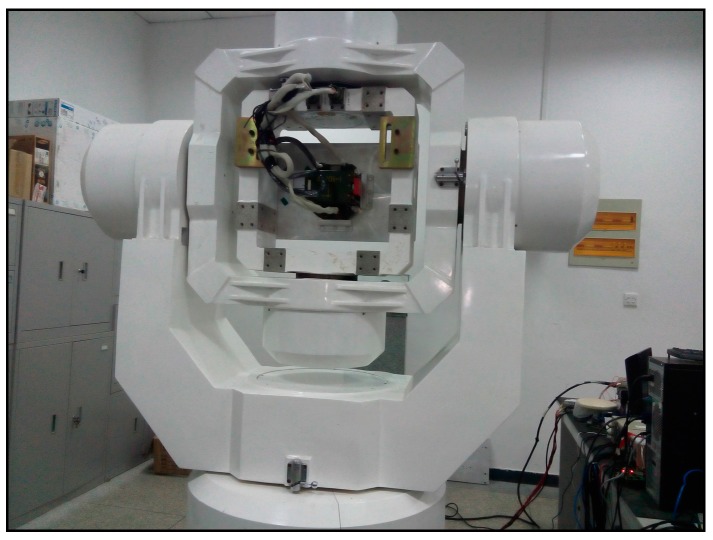
Three-axis turntable.

A turntable test is conducted to verify the dual-Euler direct approach. The three-axis turntable is shown in Figure 11. The turntable attitude is expressed in terms of positive Euler angles. For simplicity, the roll and heading keep constant, and they are zero. The pitch changes between −π/2 and +π/2 linearly and periodically, and sometimes it is kept constant at ±π/2 for a few seconds. The turntable attitude is simultaneously recorded, and it is considered to be the true value and is used as the attitude reference (denoted as True). It should be noted that for the choice of angular motions, the positive Euler angle direct approach cannot be used to estimate the attitude at each time.

The inertial sensor data are sampled at 100 Hz (*T*_1_ = 10 *ms*). The measurement of the UKF is given by ***z*** = [0,0,0]*^T^*, and the initial state vector of the filter is set as ***x*_0_** = [0,0,*θ*_0_, *γ*_0_, *ψ*_0_,0,0,0,0,0]*^T^*. Other parameter settings are as same as those specified in Section 4. The test total time is about 41 min, of which the first 5 min is used for the coarse alignment. The last 36 min is used for the fine alignment.

**Figure 12:**
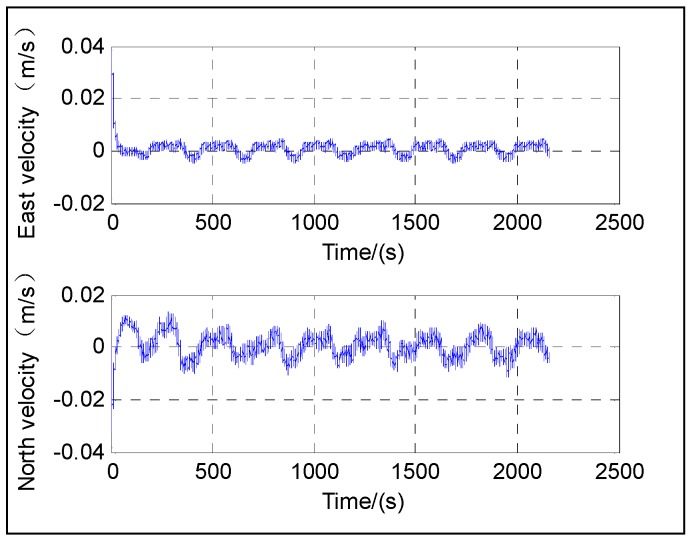
East and north velocities.

**Figure 13:**
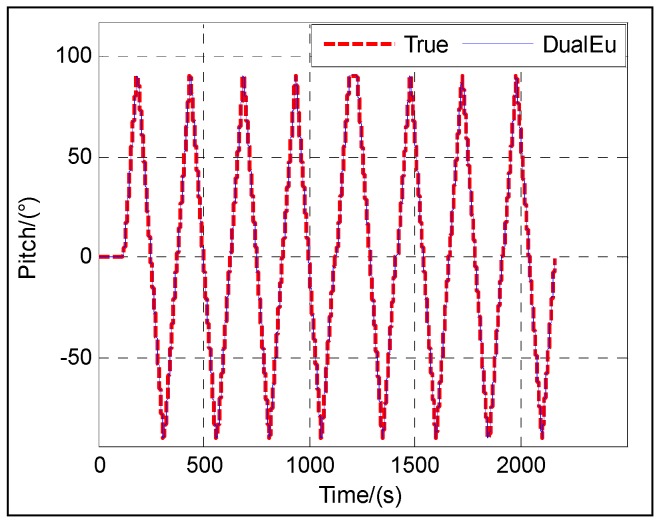
Pitch.

**Figure 14:**
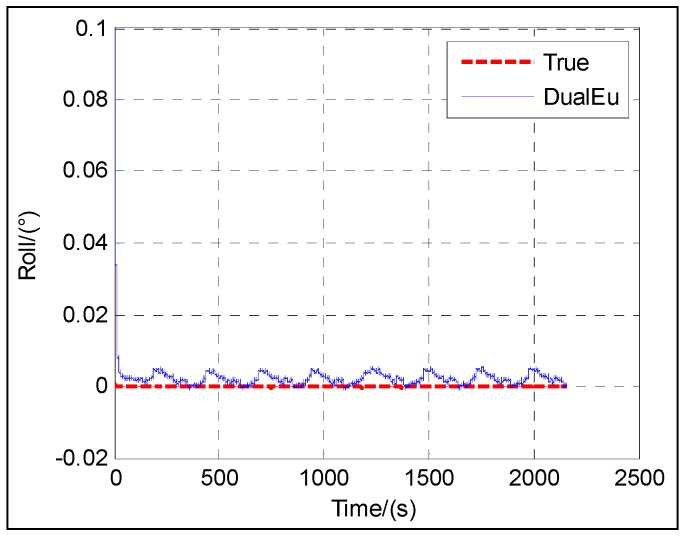
Roll.

**Figure 15:**
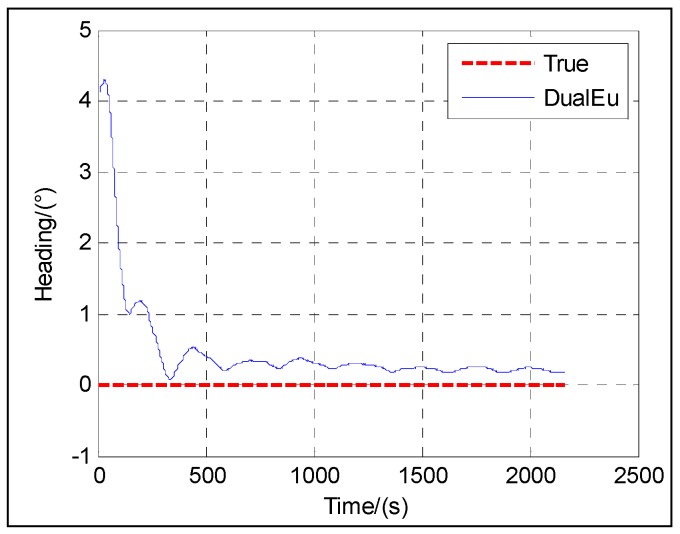
Heading.

**Figure 16:**
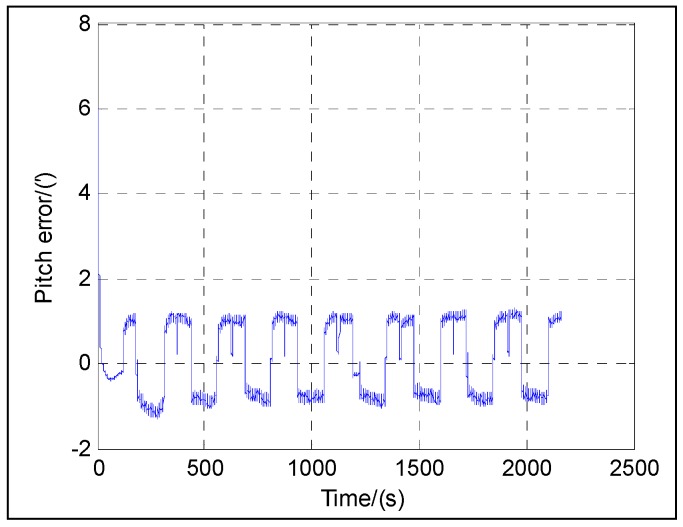
Pitch error.

**Figure 17:**
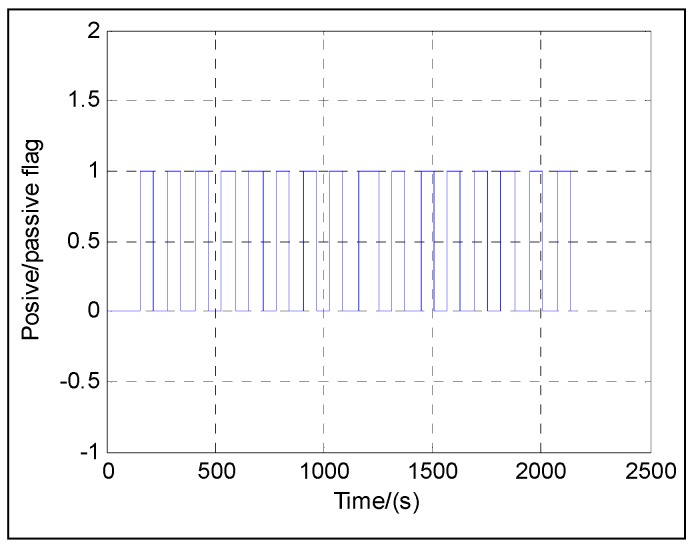
Switch flag.

The following figures give the singularity test results in a period of fine alignment using the dual-Euler direct approach (denoted as DualEu). Figure 12 shows the estimated east and north velocities. Figure 13, Figure 14 and Figure 15 show the true and estimated attitudes. Figure 16 shows the estimated Pitch angle errors, i.e., the differences between the estimated pitch and the true one. Figure 17 shows the switch flag, denoting the positive or passive Euler angle direct approach is selected.

From Figure 13 and Figure 17, it is clear that there is nearly no difference between the estimated pitch and the true one even at *θ* = ±π/2 and the positive and passive Euler angle direct approaches are used alternately according to the switch flag. The positive Euler angle direct approach is implemented if ∣*θ*∣ ≤ π/4, otherwise, the passive Euler angle direct approach is implemented. Initially, 0 ≤ *θ* ≤ π/4, the flag is set to 0, and the positive Euler angle direct approach is used. With the increasing of *θ* from 0 to π/2, once *θ* > π/4, the flag is set to 1, and the switching occurs, and the passive Euler angle direct approach is used. Then with the decreasing of *θ* from π/2 to 0, the passive Euler angle direct approach runs until *θ* ≤ π/4. Once *θ* ≤ π/4, the flag is set to 0 again, and the inverse switching occurs, and the positive Euler angle direct approach is used. The switching will be carried out in the same way subsequently. The passive Euler angle direct approach works well when the positive Euler angle direct approach is singular at *θ* = ±π/2.

The test results shown in Figure 12 illustrate the convergence of the velocity at about 20 s. From Figure 13, Figure 14, Figure 15 and Figure 16, it can be seen that the attitude converges with time. The convergence of the pitch and roll is greatly faster than that of the heading. Figure 13 and Figure 16 show the plots of the pitch and the associated error with time, respectively. The pitch converges at about 20 s, and the mean absolute value of the pitch errors is 0.86′ after 30 s. Figure 14 shows the plots of the roll with time. The roll converges at about 20 s, and the mean absolute value of the roll errors is 0.13′ after 100 s. Figure 15 shows the plots of the heading with time. The heading converges at about 300 s, and the mean absolute value of the heading errors is 15.92′ after 300 s.

According to the above singularity test results, the singularity at *θ* = ±π/2 of the positive Euler angle direct approach can be efficiently removed by means of the dual-Euler method, and the convergence speed and alignment precision can satisfy the requirement of initial alignment.

## 6. Conclusions

In this paper a direct UKF approach is proposed for aided SINSs with the following highlights: (1) the direct integration navigation solution is completely nonlinear, and it can achieve better performance than typical indirect and linear approaches. The UKF outputs the navigation information directly. The approach combines the navigation computation with the state estimation, and there is no need to process navigation computation and error correction separately. As an alternative, it can be considered as a supplement to the conventional integration navigation algorithm; (2) the system model and measurement model are conceptually simple and easy to understand, as no linear restrictions or approximations are needed, while SINS error models are derived in a complex way with many assumptions; (3) the direct approach separates the inertial sensor noise from the aid measurement noise in the measurement models, and it overcomes the cross-noise problem which exists in the SINS indirect error models; (4) the Euler angle kinematics equation is employed instead of the quaternion kinematics equation, and there is no nonlinear constraint during the filtering process. Therefore, the proposed method is easy to apply and the algorithm is simple; (5) the singularity problem, which exists in the Euler angles, can be efficiently solved by means of the dual-Euler method. The proposed approach is non-singular because the positive and passive Euler angle direct approaches operate alternately according to the switch flag to remove the singularities; (6) this direct approach can be applied to cases where it is difficult to develop linear models and use the KFs, for example, the tightly coupled DVL/SINSs [34], which do not require bottom lock or allow individual validation and characterization of the DVL beam measurements, i.e., each beam velocity is treated as a separate measurement; and the tightly coupled USBL/SINSs [35], which directly exploits the acoustic array spatial information. The direct approach can also be applied to RP, MRP and GRP, since their three parameters are uncorrelated.

## Figures and Tables

**Table 1:** Trajectory information.

Parameter	Trajectory 1	Trajectory 2	Trajectory 3
Time (min)	25	30	30
Distance (km)	11.4	10.6	14.3

**Table 2:** Position errors.

Error	Trajectory 1	Trajectory 2	Trajectory 3
Direct	1.4%	1.58%	2.36%
Indirect	2.5%	3.12%	3.9%

## References

[1] Bristeau P.J., Petit N., Praly L. (2010). Design of a navigation filter by analysis of local observability. Proceedings of the 49th IEEE Conference on Decision and Control (CDC).

[2] Bristeau P.J., Petit N. (2011). Navigation System for Ground Vehicles Using Temporally Interconnected Observers. Proceedings of the 2011 American Control Conference (ACC).

[3] Seo J., Lee H.K., Lee J.G., Park C.G. (2006). Lever arm compensation for GPS/INS/odometer integrated system. Int. J. Control. Autom..

[4] Kim S.B., Bazin J.C., Lee H.K., Choi K.H., Park S.Y. (2011). Ground vehicle navigation in harsh urban conditions by integrating inertial navigation system, global positioning system, odometer and vision data. IET Radar Sonar Navig..

[5] Hegrenaes O., Hallingstad O. (2011). Model-aided INS with sea current estimation for robust underwater navigation. IEEE J. Ocean. Eng..

[6] Liu X.X., Xu X.S., Liu Y.T., Wang L.H. (2014). Kalman filter for cross-noise in the integration of SINS and DVL. Math. Probl. Eng..

[7] Zhao L., Qiu H.Y., Feng Y.M. (2016). Analysis of a robust Kalman filter in loosely coupled GPS/INS navigation system. Measurement.

[8] St-Pierre M., Gingras D. (2004). Comparison between the unscented Kalman filter and the extended Kalman filter for the position estimation module of an integrated navigation information system. Proceedings of the 2004 IEEE Intelligent Vehicles Symposium.

[9] Gong X.L., Fan W., Fang J.C. (2014). An innovational transfer alignment method based on parameter identification UKF for airborne distributed POS. Measurement.

[10] Enkhtur M., Cho S.Y., Kim K.H. (2013). Modified unscented Kalman filter for a multirate INS/GPS integrated navigation system. ETRI J..

[11] Cheng J.H., Chen D.D., Landry R.J., Zhao L., Guan D.X. (2014). An adaptive unscented Kalman filtering algorithm for MEMS/GPS integrated navigation systems. J. Appl. Math..

[12] Li W.L., Wang J.L., Lu L.Q., Wu W.Q. (2013). A novel scheme for DVL-aided SINS in-motion alignment using UKF techniques. Sensors.

[13] Julier S., Uhlmann J., Durrant-Whyte H.F. (2000). A new method for the nonlinear transformation of means and covariances in filters and estimators. IEEE Trans. Autom. Control.

[14] Julier S.J., Uhlmann J.K. (2004). Unscented filtering and nonlinear estimation. Proc. IEEE.

[15] Qi H.H., Moore J.B. (2002). Direct Kalman filtering approach for GPS/INS integration. IEEE Trans. Aerosp. Electron. Syst..

[16] Choukroun D., Bar-Itzhack I.Y., Oshman Y. (2006). Novel quaternion Kalman filter. IEEE Trans. Aerosp. Electron. Syst..

[17] Jiang Y.F., Xie B., Weng J. (2013). SINS in-motion alignment and position determination for land-vehicle based on quaternion Kalman filter. Proceedings of the Chinese Control Conference (CCC).

[18] Zhang P.F., Gu J., Milios E.E., Huynh P. (2005). Navigation with IMU/GPS/digital compass with unscented Kalman filter. Proceedings of the IEEE International Conference on Mechatronics & Automation.

[19] Kraft E. (2003). A quaternion-based unscented Kalman filter for orientation tracking. Proceedings of the Sixth International Conference of Information Fusion.

[20] Shin E.H., El-Sheimy N. (2004). An unscented Kalman filter for in-motion alignment of low-cost IMUs. Proceedings of the 2004 Position Location and Navigation Symposium (PLANS 2004).

[21] Khoder W., Fassinut-Mombot B., Benjelloun M. Quaternion unscented Kalman filtering for integrated inertial navigation and GPS. Proceedings of the 2008 11th International Conference on Information Fusion.

[22] Van Merwe R.D., Wan E.A., Julier S.I. Sigma-point Kalman filters for nonlinear estimation and sensor-fusion—Applications to integrated navigation. Proceedings of the AIAA Guidance, Navigation, and Control Conference and Exhibit.

[23] Julier S.J., LaViola J.J. (2007). On Kalman filtering with nonlinear equality constraints. IEEE Trans. Signal Proc..

[24] Crassidis J.L. (2006). Sigma-Point Kalman filtering for integrated GPS and inertial navigation. IEEE Trans. Aerosp. Electron. Syst..

[25] Georgy J., Noureldin A., Goodall C. (2012). Vehicle navigator using a mixture particle filter for inertial sensors/odometer/map data/GPS integration. IEEE Trans. Consum. Electr..

[26] Shabani M., Gholami A., Davari N. (2015). Asynchronous direct Kalman filtering approach for underwater integrated navigation system. Nonlinear Dyn..

[27] Shuster M.D. (1993). A survey of attitude representations. J. Astronaut. Sci..

[28] Schaub H., Junkins J.L. (1996). Stereographic orientation parameters for attitude dynamics: A generalization of the Rodrigues parameters. J. Astronaut. Sci..

[29] Singla P., Mortari D., Junkins J.L. (2004). How to avoid singularity when using Euler angles. Proceedings of the AAS/AIAA 14th Space Flight Mechanics Meeting.

[30] Mortari D., Angelucci M., Markley F.L. (2000). Singularity and attitude estimation. Proceedings of the AAS/AIAA 10th Space Flight Mechanics Meeting.

[31] Qun L.H., Hou S. (2013). Research on control aircraft trajectory in simulating game scene based on the doubler Euler method. J. Appl. Sci..

[32] Wu B., Chen P., Hu Y.J., Wang C.L. Research on attitude singularity problem of small tail-sitter aircraft. Proceedings of the 4th International Conference on Frontiers of Manufacturing Science and Measuring Technology (ICFMM 2014).

[33] Yoon S. A study on optimal switching angles in dual-Euler method. Proceedings of the AIAA Modeling and Simulation Technologies Conference and Exhibit.

[34] Miller P.A., Farrell J.A., Zhao Y.Y., Djapic V. (2010). Autonomous underwater vehicle navigation. IEEE J. Ocean. Eng..

[35] Morgado M., Oliveira P., Silvestre C. (2013). Tightly coupled ultrashort baseline and inertial navigation system for underwater vehicles: An experimental validation. J. Field Robot..

